# The influence of a mineral oil cationic nanoemulsion or perfluorohexyloctane on the tear film lipid layer and higher order aberrations

**DOI:** 10.1371/journal.pone.0279977

**Published:** 2023-01-18

**Authors:** Kirsten Julia Habbe, Andreas Frings, Amr Saad, Gerd Geerling

**Affiliations:** Department of Ophthalmology, Heinrich Heine University, Düsseldorf, Germany; Xiamen University, CHINA

## Abstract

**Purpose:**

To prospectively assess the effect of a single and regular application of either a cationic nanoemulsion of mineral oil (CN) or perfluorohexyloctane (F6H8) on the lipid layer of the tear film and higher order aberrations (HOA) in patients with Dry Eye Disease (DED).

**Methods:**

Fifty-seven patients with a lipid layer thickness (LLT) ≤ 75 interferometric colour units (ICU) were included in the study. In group A (20 patients) the effect of a single drop of F6H8 or CN on HOA and LLT was assessed immediately after application and up to two hours later. For long term effects (Group B) 37 patients applied CN or F6H8 five times a day for 12 weeks. Measurement of LLT, HOA, non-invasive-tear-break-up-time (NIBUT) and meibography were assessed prior to as well as at 4 weeks and 12 weeks after initiation of treatment. Our study is registered in the “German Clinical Trials Register” under the trial number: DRKS00028696.

**Results:**

CN led to an increase of the LLT from 46.8 ± 16.9 ICU to 76.3 ± 23.5 ICU (p = 0.021) and to an increase of HOA from 0.43 ± 0.06 μm to 0.48 ± 0.08 μm immediately after application (p = 0.027). There was no correlation between the increase of LLT and HOA (r = -0.04; p = 0.90). In group B an increase of LLT was observed in the F6H8 group from 45.8 ± 8.8 ICU at baseline to 66.7 ± 19.5 ICU at 12 weeks (p = 0.002). No changes of HOA were measured throughout the observation period in group B. After 12 weeks CN increased NIBUT from 9.9 ± 5.3 seconds to 15.5 ± 5.6 seconds (p = 0.04). F6H8 increased NIBUT from 12.4 ± 5.9 seconds to 16.9 ± 4.7 seconds (p = 0.02) after 12 weeks.

**Conclusion:**

CN leads to a short-term increase in LLT and HOA, but only immediately after application. In contrast F6H8 does lead to an increase of LLT after regular long-term use but has no effect on HOA. The regular application of lipid-based products does not seem to decrease the quality of vision as measured in HOA. Instead, CN and F6H8, both are able to stabilize the tear film after regular application.

## Introduction

Dry Eye Disease (DED) is defined as a multifactorial disease of the ocular surface characterized by a loss of homeostasis of the tear film, accompanied by ocular symptoms, in which tear film instability and hyperosmolarity, ocular surface inflammation and damage as well as neurosensory abnormalities play etiological roles [1]. Patients complain about symptoms of dryness, pain, photosensitivity and decreased quality of vision, the latter potentially being due to optical aberrations [1]. Higher order aberrations (HOA) occur in the presence of corneal deformations but also in the presence of disorders of the tear film, which are typical for DED [2].

The healthy tear film provides a smooth refractive surface which is of vital importance for optimal visual acuity [3]. Montés-Micó et al. showed that alterations of its aqueous component can result in HOA [4]. However, the tear film´s superficial lipid layer, mostly produced by the Meibomian glands, is most important for its stability as it prevents the remaining layers from evaporation [5]. In the past years Meibomian gland disease (MGD) has been found to be the main pathomechanism for evaporative DED [6]. Therefore lipid-based tear supplements have moved into the scientific focus [7].

A large number of lipid containing lubricants is now available. These can be prepared as emulsions [8]. Emulsions contain non-soluble liquid components which are dispersed within other liquids [9]. In the case of lipid-based formulations, emulsions contain oily droplets which are mixed with water [10]. To be able to mix the two different liquids, surfactants must be used to apply enough shear forces and pressure to overcome surface tension [9]. Emulsions can be differentiated by drop size and charge (e.g. cationic or anionic) [10]. Nanoemulsions can be mixed with additional components such as for example cetalkonium chloride (CKC) as cationic agent to increase adsorption to the negatively charged cell membranes of the ocular surface, thus prolonging residence time [11]. Some lipid-based products are completely free of water, e.g. ointments [10] or semifluorinated alkanes [12]. Perfluorohexyloctane (F6H8) is a semifluorinated alkane that has lipophilic activity and significant spreading abilities that reduce shear forces [12]. Due to its amphiphilic nature, F6H8 does not need to be produced as an oil-in-water emulsion but can be used as a single compound solution [12]. The purpose of this study was to assess the effect of these two tear supplements on the tear film lipid layer and HOA after short or long-term application.

## Materials and methods

### Trial design

This was a parallel-group, randomized, active-controlled trial.

### Participants, eligibility criteria, and settings

Eighty-two patients were included in this study. Two separate cohorts were recruited, one (A) to evaluate the immediate effect of a single application of two different lipid-based lubricants and a second (B) to assess the effect of a 12-week treatment with regular application five times per day.

In cohort A (short term effect) after baseline measurement one single drop of study medication was applied. Both eyes were timed separately to ensure the same interval for all measurements. In 10 controls, one eye did not receive study medication. All measurements were taken immediately, 15 and 120 minutes after the application. To avoid disturbances of the tear film that would affect the continuous measurements, we resigned from further evaluation of other tear film parameters.

In cohort B (long term effect) all participants applied the study medication 5 times per day for 12 weeks. All examinations were performed at baseline, and at 4- and 12-week follow-up as described above. They were advised to suspend the application of study medication 12 hours prior to the measurements. For cohort B an age- and sex-matched control group of healthy volunteers was recruited, which did not receive any treatment.

Inclusion criteria were a lipid layer deficit, defined as less than 75 interferometric color units (ICU) measured with the LipiView^®^ interferometer together with a pathological Ocular Surface Disease Index (OSDI) of 13 or higher. Exclusion criteria were a LLT of more than 75 ICU, an OSDI lower than 13, the application of any other eyedrops during the study period, the presence of any other previously diagnosed chronic ocular surface disease (e. g. perennial allergic conjunctivitis) and/or recent ocular surgery. Patients were advised to stop any other topical therapy at least 2 days before baseline.

The study took place at the Department of Ophthalmology at the University Hospital in Düsseldorf, Germany.

### Interventions

Participants in both cohorts were randomized to either receive a cationic nanoemulsion (CN, Cationorm^®^; Santen Pharmaceutical, Munich, Germany) that contains cetalkonium chloride, liquid paraffin, glycerol, tyloxapol, poloxamer 188, tris hydrochloride and tromethamine) or a completely non-aqueous liquid that only contains perfluorohexyloctane (F6H8), a semifluorinated alkane (SFA, Evotears^®^ Ursapharm Arzneimittel GmbH, Saarbrücken, Germany).

### Outcome measurements

All measurements followed the same protocol: First, symptoms were assessed using the Ocular Surface Disease Index (OSDI^®^). Then HOA were measured with wavefront aberrometry followed by interferometry of the lipid layer in both eyes. Non-invasive tear-break-up time (NIBUT) was also measured. Finally, meibography was performed. To minimize natural circadian fluctuations, all examinations took place between 10 a.m. and 2 p.m.

HOA were calculated from wavefront data based on the ray tracing method and corneal front surface data obtained with a rotating Scheimpflug camera (Pentacam^®^, Oculus^®^ Optikgeraete GmbH, Wetzlar, Germany). Measurements were performed under standardized mesopic conditions with identical pupil sizes and were taken 3 seconds after the last blink as suggested by Hagyo et al. [13]. We analyzed the RMS values (root mean square) of HOA. The RMS value is the standard deviation of all polynomials, representing the mean deviation of all Zernike coefficients from the ideal wavefront in a single eye [14].

Non-invasive measurements of the lipid layer thickness were performed with the LipiView^®^ interferometer (TearScience Inc.) which directly measures the LLT non-invasively [15]. A special lightning source is pointed at the tear film of the patient`s eye which is able to visualize specific color patterns that arise whenever an aqueous phase is covered by a lipid layer [16]. As a result, data are reported as interferometric color units (ICU), with one ICU representing approx. 1 nm LLT [17].

NIBUT was determined non-invasively using the Oculus Keratograph 5M (Oculus^®^ Optikgeraete GmbH, Wetzlar, Germany). To ensure measurement reproducibility, patients were asked to blink twice and then to keep their eyes open as long as possible. The measurement ended automatically as soon as there was a significant distortion of the reflection of the Placido-rings.

Meibography was also performed with the Oculus Keratograph 5M (Oculus^®^ Optikgeräte GmbH, Wetzlar, Germany). Patients were seated in front of its camera. After eversion of the upper and lower eyelid the camera was manually focused and triggered. MGD was classified according to Arita et al [16] from Grade 0 (no loss), Grade 1 (less than one third), Grade 2 (between one and two thirds) to Grade 3 (more than two thirds of MG lost).

### Sample size consideration

Sample size calculation is obtained from previous studies.

### Randomization

**Fig 1:**
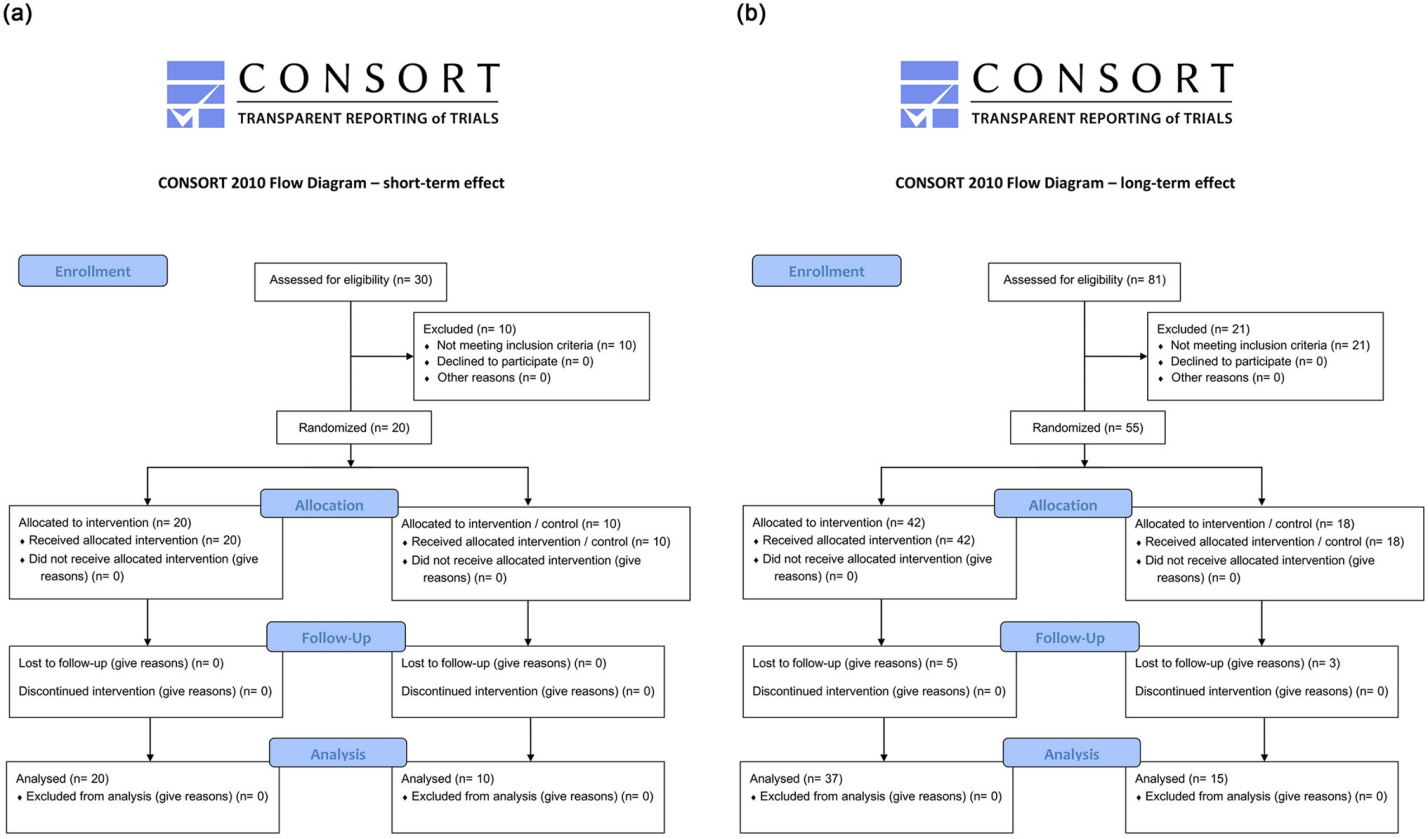
a—CONSORT Flow Diagram for short-term effect group. b—CONSORT Flow Diagram for long-term effect group.

Using a simple unrestricted system using the random generator function of Excel (Microsoft Excel 2017, Microsoft^®^, Redmond; Washington, USA) the trial statistician performed an allocation concealment. We avoided any randomization bias because the trial statistician was not involved in the recruitment process. Using this feature, patients were randomly assigned to cohorts A and B, and 10 patients were selected in cohort A whose second eye was left without any intervention to serve as a control group (Fig 1a and 1b).

### Ethics approval

This prospective interventional randomized study included patients who were referred to Dry Eye clinic of the Department of Ophthalmology, University Hospital Duesseldorf, Germany, and healthy individuals without a history of DED who served as controls. All participants gave written informed consent for pseudonymized data analysis and anonymous publication during the recruitment process. The study and consent procedure were approved by the ethics committee of the University of Duesseldorf (no. 6057R), Germany, and adhered to the tenets of the Declaration of Helsinki. Our study is registered in the “German Clinical Trials Register” under the trial number: DRKS00028696. The results reported in this study are long-term data. Our reporting of results is completely transparent, and our clinical trial is registered in detail, so the quality remains high and retrospective registration did not influence the outcome of this study. The reason why we did not register our study in a registry is because we were under the impression that our local ethics committee´s approval was sufficient. The authors confirm that all ongoing and related trials for this drug/intervention are registered.

### Statistical analysis

Statistical analysis was performed using IBM SPSS Statistics 25.0.0.2 (IBM, Armonk, NY, USA). Patient data were analyzed with the Chi-Square and the Kruskal-Wallis test. According to McAlinden et al [18], only one eye of each patient was used for the evaluation. The eye with the lower lipid layer thickness was selected for this purpose. Due to violations of assumption for a mixed ANOVA, such as data not being completely normally distributed according to the Shapiro-Wilk test, the Friedmann test and Wilcoxon signed rank test were used to compare the baseline with follow-up (FU). To compare parameters between groups at baseline and FU, a Kruskal-Wallis test and a Mann-Whitney test were applied. Each P-value was subjected to a Bonferroni adjustment. To show a possible correlation between the parameters, the Spearman correlation was chosen due to the non-parametric distribution. A P-value of 0.05 or less was defined to be statistically significant.

## Results

### Short term analysis

In total 30 patients with DED were screened. 20 patients fulfilled the inclusion criteria, had no exclusion criteria, and were randomized to receive one of the two study medications (CN-group: n = 10; 5 males; 5 females; mean age = 40.8 ± 16.9 years; F6H8-group: n = 10; 3 males; 7 females; mean age = 39.2 ± 18.5 years). 10 of the 20 patients were randomly selected whose second eye served as a control group (control: n = 10; 6 males; 4 females; mean age 41.2 ± 19.0). There were no significant differences gender (X^2^ (2) = 1.8; p = 0.4) or in age (H (2) = 0.1; p = 0.7) between all three groups (Tables 1 and 2).

**Table 1:** Gender distribution (short term analysis group).

group	male	female	total
**CN**	5	5	10
**F6H8**	3	7	10
**control**	6	4	10
**total**	14	16	30
**p-value**	0.392		

The gender distribution was calculated using the chi-square test.

CN = cationic nanoemulsion of mineral oil; F6H8 = Perfluorohexyloctane.

**Table 2:** Age distribution (short term analysis group).

group	N	mean	SD	minimum	maximum
**CN**	10	40.80	16.93	24.00	68.00
**F6H8**	10	39.20	18.53	23.00	68.00
**control**	10	41.20	19.01	23.00	68.00
**total**	30	40.40	17.57	23.00	68.00
**p-value**	0.676				

The age distribution was calculated using the Mann-Whitney test.

**CN** = cationic nanoemulsion of mineral oil; **F6H8** = Perfluorohexyloctane; **N** = number of patients; **SD** = standard deviation.

**Fig 2:**
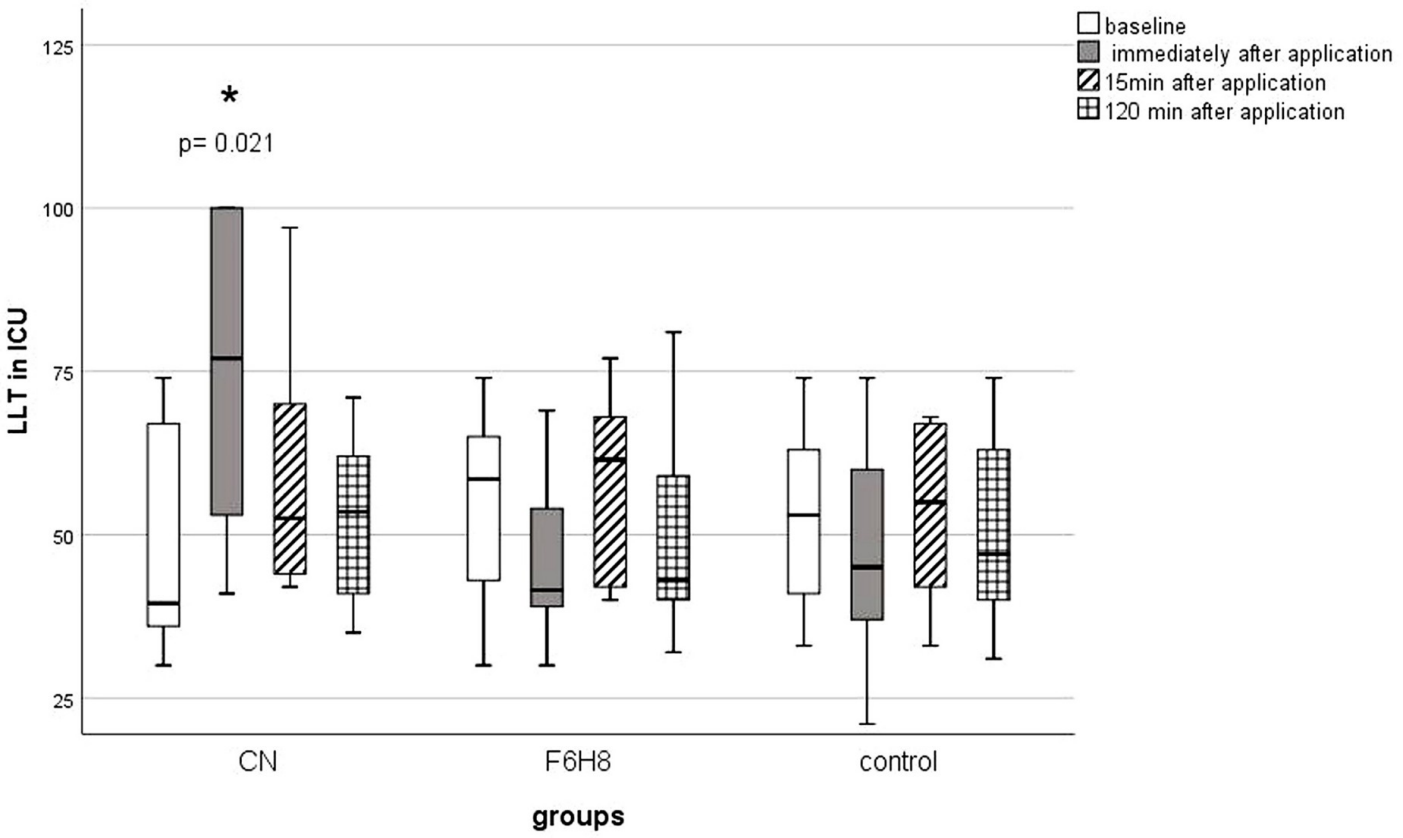
Lipid layer thickness (in ICU) at baseline and consecutive measurements. The measurement values up to 120 min after application were compared with the baseline using the Wilcoxon test. All p-values were adjusted for 3 comparisons according to the Bonferroni method. An asterisk with the corresponding p-value indicates a significant change compared to baseline measurement. ICU = Interferometric Colour Units; CN = cationic nanoemulsion; F6H8 = perfluorohexyloctane.

The LLT of the three groups did not differ at baseline (H (2) = 1.2; p = 0.5). Directly after application of CN, there was an increase of LLT from 46.8 ± 16.8 ICU to 76.3 ± 23.5 ICU (z = -2.7; p = 0.021). After 15 min (z = -1.8; p = 0.2) and 120 min (z = -1.7; p = 0.2) the LLT had returned to the baseline level (Fig 2). In comparison, there was no statistically significant change of LLT immediately after application of F6H8 (z = -2.1, p = 0.08), 15 min (z = -1.6; p = 0.3) or 120 min later (z = -1.2; p = 0.6). Controls remained unchanged as well (X^2^ (3) = 7.5; p = 0.058). Only immediately after application, the LLT of the CN group was higher than that of F6H8 (U = 13.5; z = -2.8; p = 0.018) and the control group (47.3 ± 16.4; U = 16.5; z = -2.5; p = 0.033).

**Fig 3:**
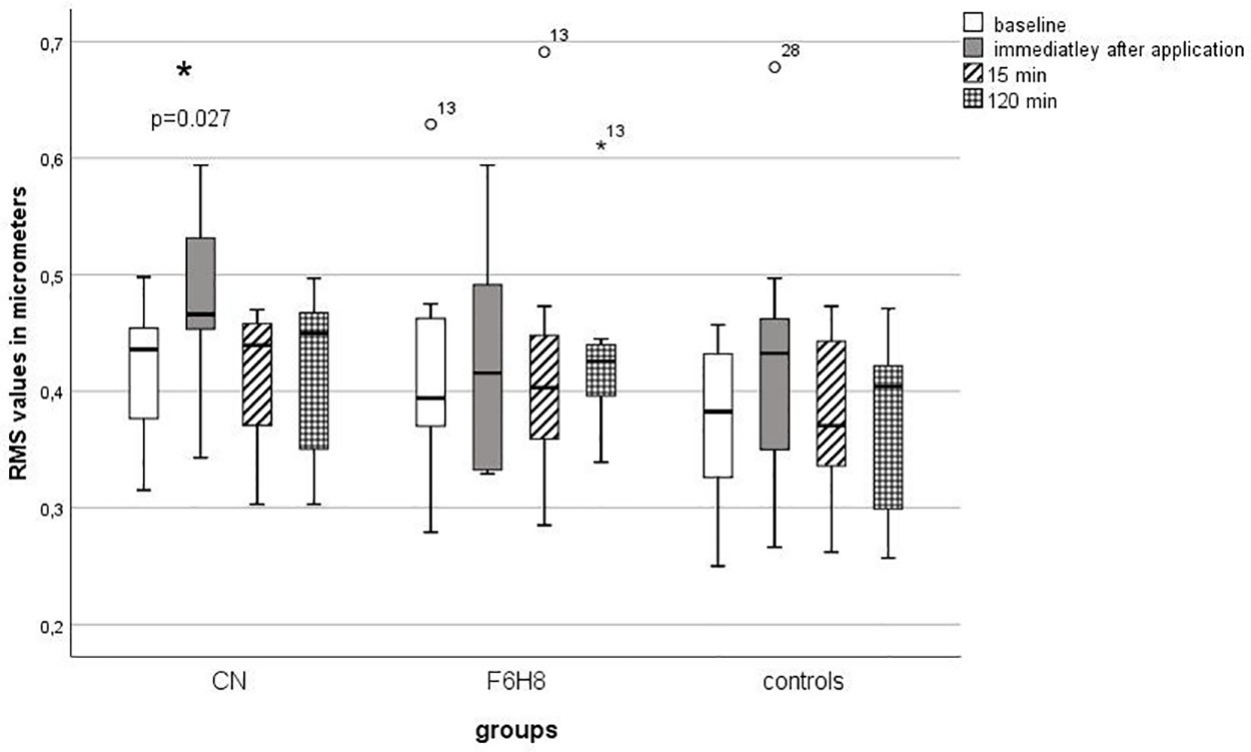
RMS values (in μm) at baseline and consecutive measurements. The measurement values up to 120 min after application were compared with the baseline using the Wilcoxon test. All p-values were adjusted for 3 comparisons according to the Bonferroni method. An asterisk with the corresponding p-value indicates a significant change compared to the baseline. RMS = Root Mean Square; μm = micrometer; CN = cationic nanoemulsion; F6H8 = perfluorohexyloctane.

At baseline, there was no difference of RMS values between the three groups (H (2) = 1.2; p = 0.5). Immediately after application, CN increased RMS (z = -2.6; p = 0.027) from 0.42 ± 0.06 μm to 0.48 ± 0.08 μm (Fig 3). The effect size was 0.58. This corresponds to a strong effect. A Spearman correlation did not show a correlation between the increase of the RMS values and the increase of LLT (r = -0.04; p = 0.9). RMS values of the CN and F6H8 group did not show a significant difference immediately after application (U = 26.0; z = -1.8; p = 0.07). The RMS values increases immediately after application of CN, however, were statistically significantly higher than the values of the control group (U = 20.5; z = -2.4; p = 0.045). After 15 min the RMS values of the CN group returned to the baseline level. In the F6H8 group, the RMS values did not change during follow-up measurements (X2 (3) = 1.7; p = 0.6). RMS values of controls also remained unchanged (X2 (3) = 2.6; p = 0.4).

There were no harms observed in either group.

### Long term analysis

In total 59 patients with DED were screened. 42 patients qualified for study inclusion, five of which were lost to follow-up or were excluded for using other tear supplements (Fig 1b). In addition, 22 healthy volunteers were screened as a control. Four of them were excluded and 3 were lost to follow-up. Ultimately 37 patients (F6H8-group: n = 18; 5 male; 13 female; mean age 24 ± 4 years; CN-group: n = 19; 8 male; 11 female; mean age 26 ± 9 years) and 15 controls (7 male; 8 female; mean age 34.5 ± 20.0 years) were included into the study. There were no significant differences in gender (X^2^ (2) = 1.4; p = 0.5) or in age (H (2) = 1.5; p = 0.5) between all three groups (Tables 3 and 4).

**Table 3:** Gender distribution (long term analysis group).

group	male	female	total
**CN**	8	11	19
**F6H8**	5	13	18
**control**	7	8	15
**total**	20	32	52
**p-value**	0.496		

The gender distribution was calculated using the chi-square test.

**CN** = cationic nanoemulsion of mineral oil; **F6H8** = Perfluorohexyloctane.

**Table 4:** Age distribution (long term analysis group).

group	N	mean	SD	minimum	maximum
**CN**	19	26.47	9.33	21.00	63.00
**F6H8**	18	24.22	4.12	21.00	37.00
**control**	15	34.53	20.01	18.00	72.00
**total**	52	28.02	12.84	18.00	72.00
**p-value**	0.469				

The age distribution was calculated using the Kruskal-Wallis test.

**CN** = cationic nanoemulsion of mineral oil; **F6H8** = Perfluorohexyloctane; **N** = number of patients; **SD** = standard deviation.

**Fig 4:**
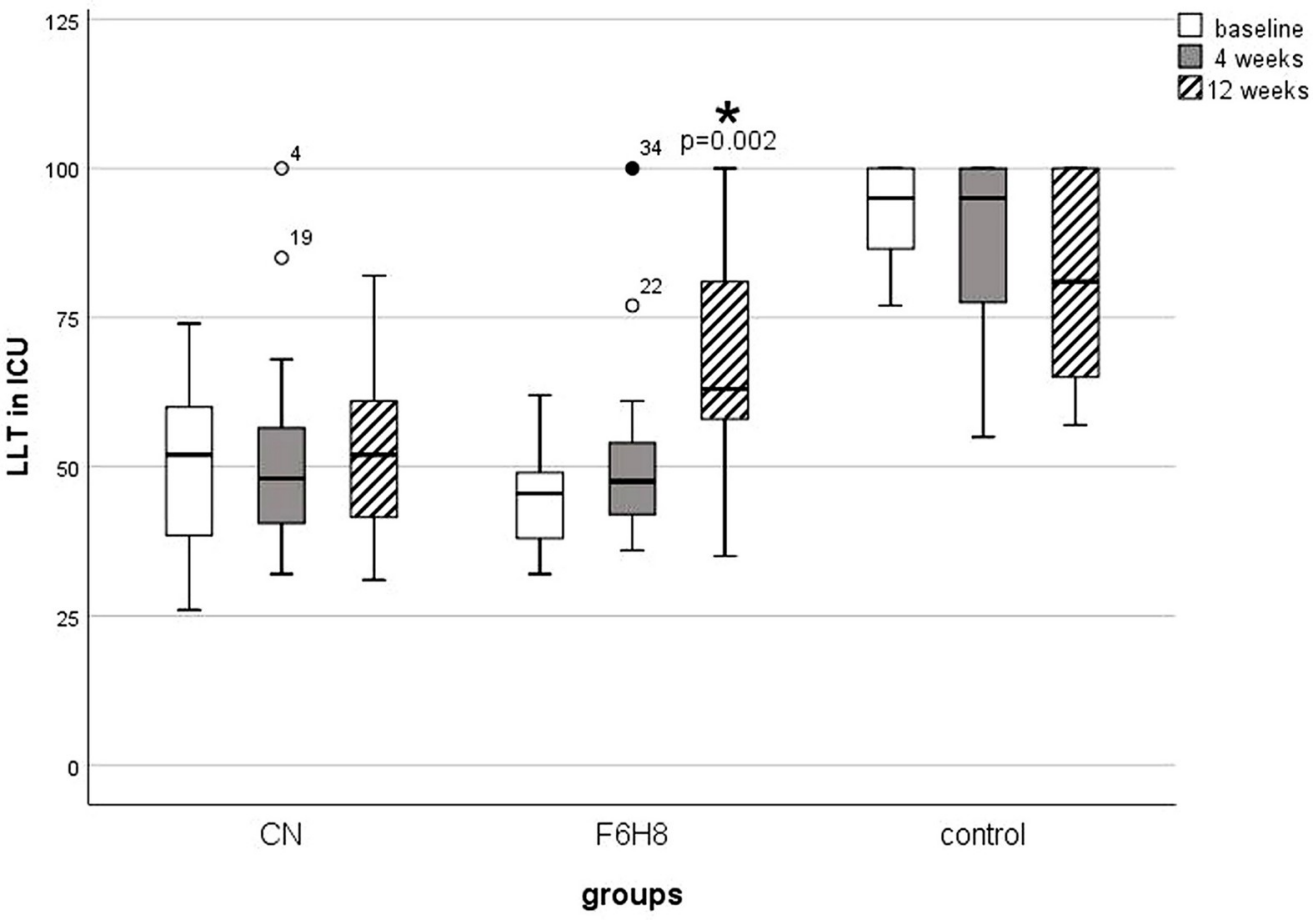
Lipid layer thickness (in ICU) at consecutive measurements. The LLT at 4 and 12 weeks were compared to the LLTat baseline using the Wilcoxon test. All p-values were adjusted for 2 comparisons according to the Bonferroni method. An asterisk with the corresponding p-value indicates a significant change compared to the baseline. ICU = Interferometric Colour Units; CN = cationic nanoemulsion; F6H8 = perfluorohexyloctane.

At baseline LLT of the CN and the F6H8 group were not significantly different (U = 135.0; z = -1.9; p = 0.8), but the LLT of the control group was higher than that of the CN (U = 0.0; z = -4.9; p <0.01) and the F6H8 group (U = 0.0; z = -4.8; p <0.01). Over the course of 12 weeks LLT (Fig 4) statistically did not change significantly either in the CN group (X2 (2) = 0.3; p = 0.8) or in the control group (X2 (2) = 3.8; p = 0.1). F6H8 increased the LLT from 45.8 ± 8.7 ICU at baseline to 66.7 ± 19.5 ICU after 12 weeks (z = -3.4; p = 0.002). The effect size was r = 0.56. This corresponds to a strong effect. While LLT of the F6H8 group was not different from either the CN group (U = 93.0; z = -2.4; p = 0.054) nor the control group after 12 weeks (U = 76.5; z = -2.1; p = 0.09), LLT of the CN group was still significantly lower than in the control group (U = 29.5; z = -3.9; p <0.001).

**Fig 5:**
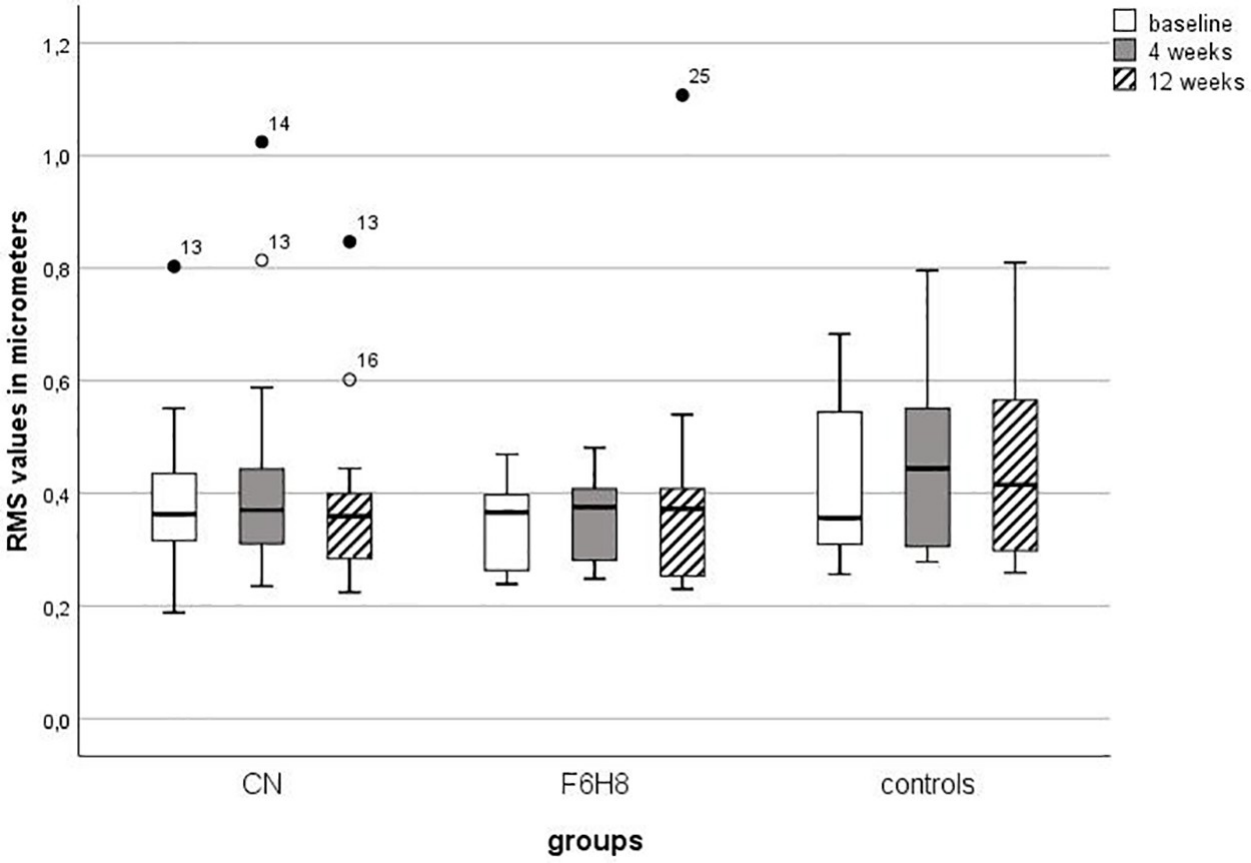
RMS values (in μm) at baseline and consecutive measurements. The RMS values at 4 and 12 weeks were compared with the values of the baseline using the Wilcoxon test. All p-values were adjusted for 2 comparisons according to the Bonferroni method. An asterisk with the corresponding p-value indicates a significant change compared to the baseline. RMS = Root Mean Square; μm = micrometer; CN = cationic nanoemulsion; F6H8 = perfluorohexyloctane.

Higher order aberrations as measured by the RMS values did not differ at baseline (H (3) = 3.0; p = 0.4) between the three groups. Neither the application of CN (X2 (2) = 0.7; p = 0.7) nor of F6H8 (X2 (2) = 1.0; p = 0.6) lead to a change in the RMS values within a 12-week observation period (Fig 5). The values of the control group also remained unchanged (X2 (2) = 3.3; p = 0.2).

**Fig 6:**
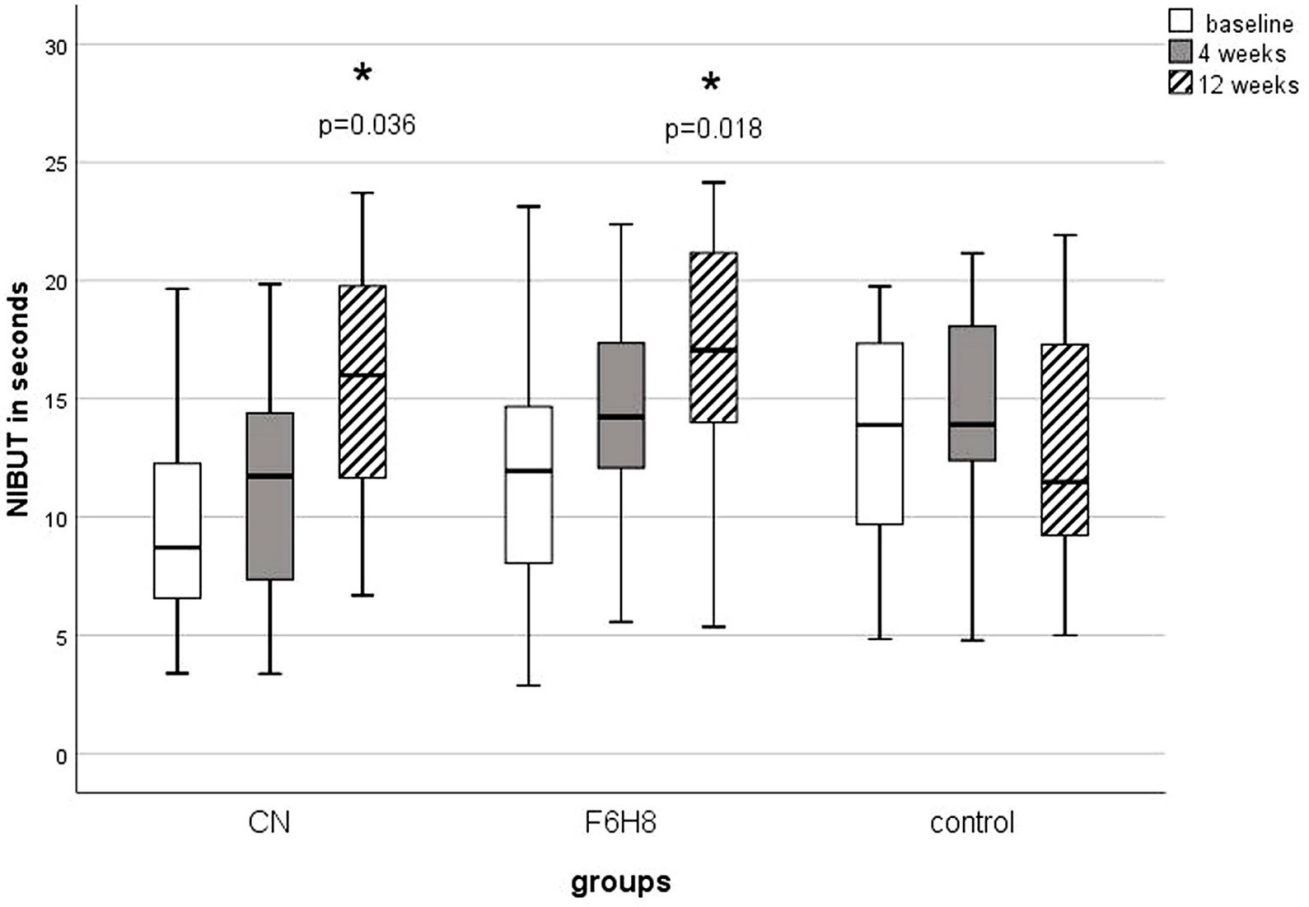
NIBUT at baseline and consecutive measurements. The NIBUT at 4 and 12 weeks were compared with the NIBUT of the baseline using the Wilcoxon test. All p-values were adjusted for 2 comparisons according to the Bonferroni method. An asterisk with the corresponding p-value indicates a significant change compared to the baseline. NIBUT = non-invasive tear break-up time; CN = cationic nanoemulsion; F6H8 = perfluorohexyloctane.

At baseline the NIBUT between the three groups did not differ significantly (H (2) = 4.7; p = 0.09). After 4 weeks there was no statistically significant difference between the groups (H (2) = 4.8; p = 0.09). After 12 weeks CN increased NIBUT statistically significant from 9.9 ± 5.3 seconds at baseline to 15.5 ± 5.6 seconds (z = -2.4; p = 0.04). The effect size was r = 0.4. This represents a medium effect. F6H8 increased NIBUT from 12.4 ± 5.9 seconds at baseline to 16.9 ± 4.7 seconds (z = -2.6; p = 0.02) after 12 weeks (Fig 6). The effect size was r = 0.4. This corresponds also a medium effect. The control group remained unchanged at all follow-ups (X2 (2) = 3.6; p = 0.1). A group comparison after 12 weeks showed that the NIBUT of the F6H8 group was significantly higher than that of the control group (U = 68; z = -2.4; p = 0.045). Applying CN led to no statistically significant changes after 12 weeks compared to control (U = 102.0; z = -1.4; p = 0.5).

**Fig 7:**
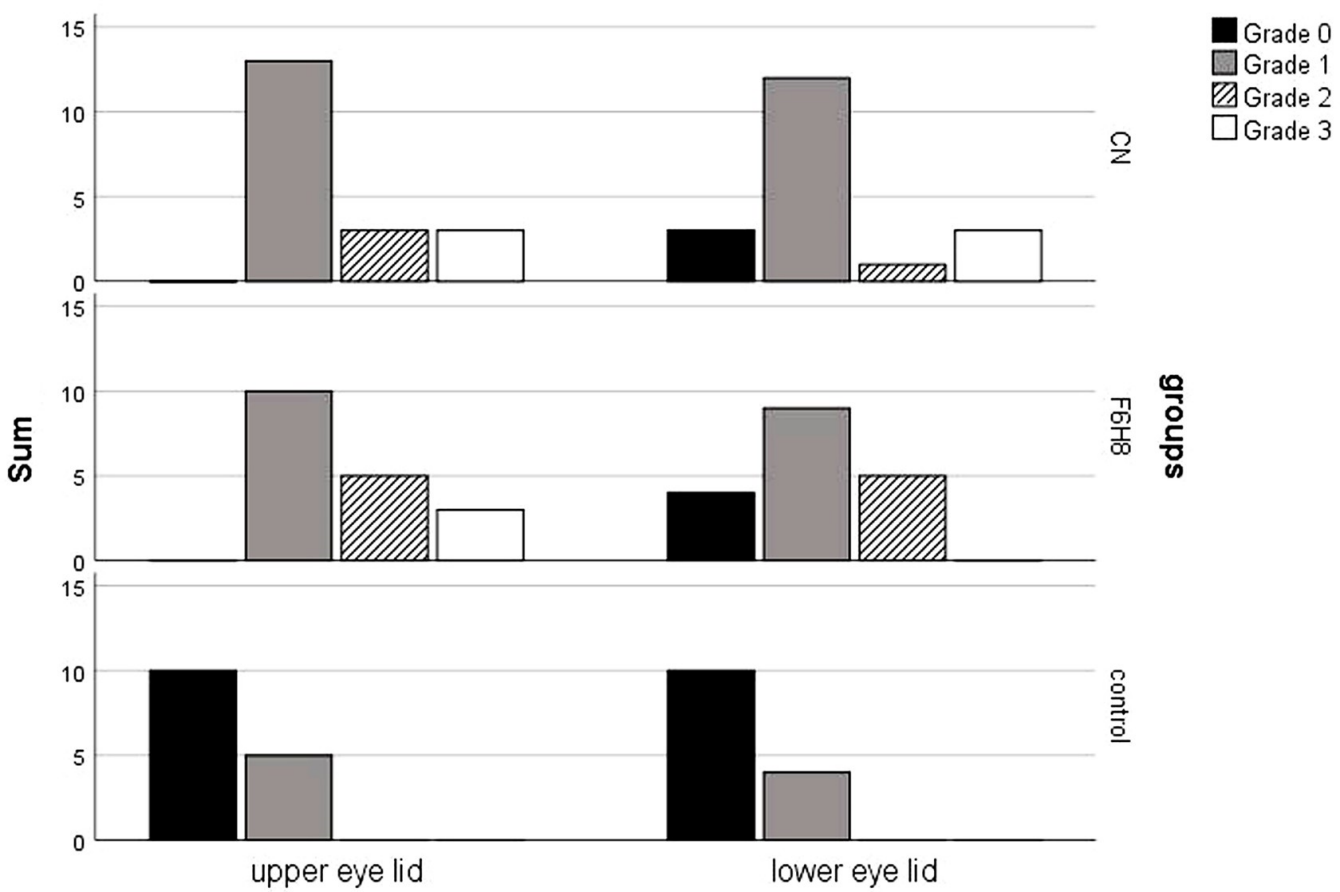
Meibomian gland scores of the upper and lower eyelid. Grade 0: No loss of Meibomian glands; Grade 1: Loss of Meibomian glands less than one third of the total density on the eyelid; Grade 2: Loss between one and two thirds of the total density of the glands on the eyelid; Grade 3: Loss of glands is greater than two-thirds of the total density of glands on the eyelid; MGD = Meibomian gland dysfunction; CN = cationic nanoemulsion; F6H8 = perfluorohexyloctane.

Both interventional groups had a comparably increased MGD score (Fig 7) of the upper (U = 160.0; z = -0.4; p = 1.0) and the lower eyelid (U = 164.5; z = -0.2; p = 1.0) at baseline. Controls had a significantly lower score at baseline (p <0.01). One study participant refused the elevation of the eyelid.

There were no harms observed in either group.

## Discussion

Previous studies have shown that HOA not only occur because of irregularities of the cornea, but also because of disorders of the tear film [2]. To date, there is contradictory evidence as towards the influence of different tear supplements on HOA and seems to depend on their main lubricant component. An improvement of HOA has been reported after application of 0.25% polyethylene glycol and / or hyaluronic acid 0.1% [19,20] while HOA increased in dry and healthy eyes after application of hypromellose (0.3%) [21]. This effect has been shown to depend on the viscosity, whereas more viscous eye drops induce higher short-term HOA [22]. The influence of the lipid layer on HOAs has not yet been thoroughly studied while more lipid-containing eye drops become readily available [7]. The objective of this work was to assess the effect of two lipid layer active lubricants on LLT and HOA.

Our study showed that LLT and HOA increase immediately after application of a cationic nanoemulsion of lipids (CN), although no correlation was found between the two parameters. Application of the lipophilic semifluorinated alkane (SFA), F6H8, did not increase the LLT. The increase of LLT shortly after application of CN is in accordance with literature [11]. The increase of HOA despite the lack of a correlation between LLT and HOA can be explained by a change of tear film dynamics immediately after application. It is known that a single eye drop—independent from its composition—changes the dynamics of the tear film up to its disruption and thus induces HOA [23]. Some authors also report a connection between the increased tear volume and HOA after application [23].

By contrast, F6H8 did not increase of HOA immediately after application. Agarwal et al. used a high-speed camera to visualize the impact of F6H8 on the tear film surface [24]. This showed that F6H8 directly merges with the tear film without causing any irregularities [24]. The observation that a single application of F6H8 does not change LLT is in line with a study by Schmidl et al. and can be explained by a technical limitation of the interferometer used [25]. LipiView^®^ can distinguish the lipid layer from the rest of the tear film because of its different refractive index [25]. Since F6H8 and water have a similar refractive index, both mix into a transparent fluid [26] leading to difficulties in distinguishing the different layers of the tear film [25]. The increase of LLT after regular application of F6H8 has also been described previously [24,25] and has been attributed to a positive effect on meibum quality [12].

Obstruction of meibomian ducts, hyperkeratinization and increased viscosity are major drivers in the pathogenesis of MGD [27]. SFAs such as F6H8 have the capability to dissolve other lipids [28] and have been applied to remove residues of silicone oil tamponades from intraocular lenses [29]. Steven et al suggest that F6H8 may dissolve pathologically altered meibum in meibomian gland orifices and hence “unplug” the MG ductules, resulting in an increased LLT [12]. Thus, the increase of LLT at 12 weeks is likely to be caused by dissolved meibum and not by an accumulation of F6H8. Despite the significant increase in LLT after 12 weeks of F6H8, there was no change in HOA. Mihaltz et al also found no effect of eye drops containing lipids or 0.2% hyaluronic acid on aberrations over a period of three months although a subgroup of more severe MGD patients showed some benefit on aberrations after 3 months [30]. Since we used a different scoring system for MGD, direct comparison of the results is limited.

Considering the significant improvement of LLT, it is still surprising that we could not measure any change in aberrations. Montés-Micó et al. postulated that the lack of tears does not necessarily lead to higher aberrations in DED [4]. A uniform reduction of the tear film seems to have little influence on the image quality [4]. However, if local irregularities occur in the precorneal tear film, this can negatively affect the optical image quality in terms of HOA [4]. The instability of the tear film and its premature break-up are usually considered to be the root cause of these local fluctuations [4]. Nevertheless, it is difficult to compare studies in this context, as the type of eye drops used, the severity of DED, the time between the last application, the number of applications and the measuring devices are inconsistent.

F6H8 and CN increased the tear-break-up time (TBUT), which is in line with literature [12,31]. While there are several other studies which demonstrate a correlation between LLT and TBUT [32], CN increased TBUT without prior increase of LLT. However, Finis et al [17] did neither find a significant association between the LLT measured with the LipiView^®^ and the NIBUT measured with the Oculus keratograph, the same devices that were used in the current study. While this–at first glance–may be surprising, many studies showing a correlation between LLT and TBUT used the installation of fluoresceine to measure TBUT, whereas we used a non-invasive method (NIBUT). Furthermore, Zhao et al. did not find a correlation between LipiView^®^ measurements and fluoresceine-tear-break-up-time (FTBUT) [33]. In addition, FTBUT and NIBUT are considered to measure different phenomena [34]. More precisely, NIBUT not only seems to detect instability of the lipid layer, but also a lack of mucin and the aqueous phase of the tear film [34]. Aside from the lipid layer, mucin also plays a vital role for the stability of the tear film [35]. Apart from CKC and oil, CN does also contain water and glycerol [36]. Due to this composition CN might not only have the potential to support the lipid phase but also the aqueous and mucinous phase of the tear film [36]. The long residence time of the nanoemulsion and the augmentation of all tear film layers might be the reason for the elongation of NIBUT with CN in contrast to F6H8 that mainly targets the lipid layer [11].

There are limitations to our study. First, the integrity and stability of the tear film are dependent on many factors, including exogenous ones. The tear film is influenced by the sequence of examinations (which was standardized in our study), weather [37], hormones [27] or a long presence in an air-conditioned environment [38] and even by gravitational effects [39]. Hence, the tear film quality can fluctuate even during measurement of aberrations, particularly after a blink and in DED [40]. After blinking, aberrations occur after an average of 2.9 seconds as a sign of premature tear break up, while these are found in healthy individuals only after 6.1 seconds [41]. Ridder et al have explained contradictory effects of lubricants on aberrations in several studies with differences in the point of time of the measurement after the blink [42].

Second, different devices for measuring HOA can only be compared to a limited extent. In a study that compared several aberrometers, the Visual Function Analyzer (Tracey Technologies, Houston Texas, USA) deviated significantly from other aberrometers in measuring the RMS values [43]. The device used in this study (Pentacam^®^) produced similar but different RMS values compared with another aberrometer [44] when using elevation data and not ray tracing [44]. While the use of the Pentacam^®^ is a limitation of our study, the device used is still able to determine higher order aberrations by ray tracing and to provide high-precision wavefront data [45].

Third, in cohort B the control group consisted of healthy individuals without DED, with a higher (i.e. normal) lipid layer thickness of > 75 ICU. While this limits comparison of the three groups at baseline, it also helped to demonstrate that F6H8 leads to normalization of the LLT.

Fourth, the presented results are based on this specific population, and future studies (on other populations) are warranted to justify the hypothesis.

In conclusion, the significant change in the lipid layer of the tear film caused by application of the lipid-based tear supplements studied, had no influence on higher-order aberrations in short- and long-term applications and thus should not have a negative impact on visual quality.
